# In vitro, ex vivo, instrumental and clinical evaluation of a topical cream on the signs of periorbital ageing

**DOI:** 10.1111/ics.12987

**Published:** 2024-08-09

**Authors:** Alexandra D'Arcangelis, Sayantani Goswami Chatterjee, Isabel Diaz, Sabine Guehenneux, Jin Namkoong, Joanna Wu

**Affiliations:** ^1^ Skin Research and Innovation, Global Personal Care and Skin Health R&D Colgate‐Palmolive Company Piscataway New Jersey USA; ^2^ Dermal Clinical Research Colgate‐Palmolive Company Piscataway New Jersey USA; ^3^ Laboratoires Filorga Paris France

**Keywords:** cosmetic, hydration, periocular ageing, puffiness, wrinkles

## Abstract

**Objective:**

Periorbital skin ageing signs are multidimensional, highly visible and a concern for many. We evaluated the potential efficacy of an eye cream to diminish these signs.

**Methods:**

Biological markers associated with ageing, barrier function and homeostasis were analysed in vitro to determine the effects of topically applied eye cream, compared to those of a placebo using human skin tissue models and/or explants. Collagen IV, elastin and bone morphogenic protein 4 (BMP4) expression was investigated by immunohistochemical labelling, while filaggrin, kallikrein 7 (KLK7) and HB‐EGF were evaluated by RT‐qPCR. IL‐1α and melanin levels in darkly pigmented skin models were also quantified. The protective effect of the cream on glycation was assessed by a non‐enzymatic assay. Finally, the benefits of twice‐daily applications of the eye cream for 56 days were instrumentally and clinically evaluated on 33 women.

**Results:**

Only the eye cream, not the placebo, stimulated collagen IV and BMP4 protein expression, as well as increased elastin fibre length. It also led to higher HB‐EGF, filaggrin and KLK7 mRNA levels. The placebo and the eye cream did not induce changes in IL‐1α and melanin levels, but both reduced non‐enzymatic glycation. When assessing the in vivo effects of the cream, short‐term results indicated skin hydration, transepidermal water loss (TEWL) and skin profilometry improvement within 15 min. Instrumental evaluations of wrinkles showed a reduction after 7 days, which was clinically perceivable after 28 or 56 days. The eye‐opening angle and eyelid sagging also improved after seven and 28 days, respectively. Finally, dark circles became lighter within 7 days (instrumental measurement) or 28 days (clinical assessment).

**Conclusion:**

The instrumental and clinical evaluations revealed that the eye cream reduced all periorbital ageing signs evaluated. Its effects are supported by the in vitro and ex vivo analyses of molecular markers.

## Graphical abstract

The topical application of the periorbital eye cream onto skin explants positively influences several age‐related skin markers. The instrumental and clinical evaluation of subjects who applied it for 56 days shows short‐ and long‐term improvement in periorbital signs of ageing.

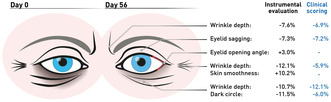

## Résumé

**Objectif:**

Les signes de vieillissement de la peau périorbitaire sont nombreux, très visibles et préoccupent de nombreuses personnes. Nous avons évalué l'efficacité potentielle d'une crème pour les yeux pour atténuer ces signes.

**Méthodes:**

Les marqueurs biologiques associés au vieillissement, à la fonction barrière et à l'homéostasie de la peau ont été analysés in vitro pour évaluer l'efficacité d'une crème pour les yeux appliquée localement. Ces effets ont été comparés à ceux d'un placebo, sur des modèles et/ou des explants de tissus cutanés humains. L'expression du collagène IV, de l'élastine et de la protéine morphogénique osseuse 4 (BMP4) a été étudiée par marquage immunohistochimique. Celle de la filaggrine, de la kallikréine 7 (KLK7), et du HB‐EGF par RT‐qPCR. Les niveaux d'IL‐1α et de mélanine dans un modèle de peau pigmentée ont également été quantifiés. L'effet protecteur de la crème sur la glycation a été évalué par un test non enzymatique. Enfin, les bénéfices d'une application biquotidienne de la crème pour les yeux ont été évalués instrumentalement et cliniquement sur 33 femmes pendant 56 jours.

**Résultats:**

Seule la crème pour les yeux a stimulé l'expression du collagène IV et de BMP4 en comparaison avec le placebo. La crème est aussi la seule à augmenter la longueur des fibres d'élastine. Elle a également entraîné une augmentation des niveaux d'ARNm de HB‐EGF, de la filaggrine et de KLK7. Le placebo et la crème pour les yeux n'ont pas modifié les niveaux d'IL‐1α et de la mélanine, mais ont tous deux réduit la glycation non enzymatique. Lors de l'évaluation des effets in vivo, les résultats à court terme ont montré une amélioration de l'hydratation de la peau, de la Perte Insensible en Eau (PIE) et du profil de la peau en 15 min. Les évaluations instrumentales de la profondeur des rides ont indiqué une réduction après 7 jours d'application, réduction cliniquement perceptible après 28 ou 56 jours. La crème périorbitale induit également une amélioration de l'angle d'ouverture des yeux et de l'affaissement des paupières respectivement après 7 et 28 jours. Enfin, les cernes sont devenus plus clairs après 7 jours (mesure instrumentale) ou 28 jours (évaluation clinique).

**Conclusion:**

Les évaluations instrumentales et cliniques ont révélé que la crème pour les yeux réduisait tous les signes de vieillissement périorbitaires évalués. Ses effets sont confirmés par les analyses in vitro et ex vivo des marqueurs moléculaires.

## INTRODUCTION

The eyes and periorbital region are essential features of facial attractiveness. They play an integral role in social interactions, as a vehicle for non‐verbal communication and to convey emotions. They are also a major attribute in estimating a person's age [1, 2]. Yet, this is the region of the face that ages first, with visible signs already appearing in the late twenties and early thirties [3, 4].

As with the rest of the skin, periorbital ageing mainly results from intrinsic ageing, the chronologically and genetically determined degenerative decline in physiological functions. Being a skin region exposed to the environment, age‐induced alterations are further aggravated by extrinsic ageing (UV irradiation, blue light, pollutants, lifestyle, etc.). Periorbital skin is particularly susceptible to these assaults due to its unique characteristics. Indeed, it is the thinnest skin on the face [5]. It is also subjected to intense mechanical stress, with around 10 000 lid beats daily, and the influence of facial expressions, including eye squinting. Further, the periorbital anatomy makes this skin especially susceptible to pollutants, as palpebral folds can accumulate them, increasing their deleterious effects.

Ageing, therefore, leads to numerous alterations [3, 6], including weaker barrier function and dryer skin. Increased skin laxity leads to drooping, even hooding, of the upper lids and falling brows. It also results in the appearance of bags below the lower lids. Dynamic wrinkles also appear, leading to crow's feet and wrinkles at the level of the lower lid. These wrinkles could be aggravated by the increased eye dryness that leads to more frequent blinking. Canthal tendons loosen, which may cause smaller eye and/or scleral show [7]. Another contribution to periorbital ageing is decreased facial fat and increased laxity of the structures securing its positioning. As a result, fat pads form, and the tear trough becomes more prominent. Cheek volume loss further aggravates these changes. Finally, infraorbital hyperpigmentation may appear. The causes of this phenomenon, known as dark circles, are not fully understood and may result from postinflammatory hyperpigmentation or the extravasation and accumulation of haemoglobin breakdown products [8, 9]. Fatigue, skin sagging and fat accumulation will contribute to its worsening.

Due to the widespread attention to skin sagging, removing excess tissue has been a frequent surgical procedure. This is particularly true for eyelids, explaining why blepharoplasty was still the first most frequent facial cosmetic surgical procedure in the USA in 2022 [10]. Several minimally invasive alternatives are available to address the multiple manifestations of periorbital ageing. With over 9.6 million procedures, botulinum toxin, hyaluronic acid fillers, laser skin resurfacing and intense pulse light treatments are, by far, preferred alternatives [10]. The industry also puts tremendous effort towards developing cosmetics that may slow, stop, or reverse ageing signs. Yet, most efforts are focused on treating only one or a few aspects of skin ageing, the most frequent being wrinkles. Several compounds have been shown to improve at least some of the multiple signs observed in the periorbital region [11, 12, 13, 14, 15, 16, 17, 18]. Perhaps formulating several active ingredients in combination would lead to more pronounced efficacy. In this study, we assessed one such potential solution. Its evaluation relied on in vitro analyses of molecular markers in human skin tissue models. The effects of topical applications on ex vivo skin explants were also analysed. Finally, a clinical evaluation was conducted to assess its effects in vivo.

## MATERIALS AND METHODS

### Topical treatment

The test product is an anti‐ageing eye contour cosmetic cream (Laboratoires Filorga Cosmétiques, Paris, France) containing the following INCI ingredients: Aqua (Water, Eau), Glycerin, Propanediol, Caprylic/Capric Triglyceride, Squalane, 1,2‐Hexanediol, C12‐16 Alcohols, *Imperata cylindrica* Root Extract, *Albizia julibrissin* Bark Extract, Hydrogenated Lecithin, Palmitic Acid, Hydroxyethyl Acrylate/Sodium Acryloyldimethyl Taurate Copolymer, *Calanthe discolour* Extract, Sodium Polyacrylate Starch, Behenyl Beeswax, Stearyl Beeswax, Parfum (Fragrance), O‐Cymen‐5‐Ol, Citric Acid, *Caesalpinia spinosa* Fruit Extract, Hydrogenated Vegetable Oil, Adenosine, Polysorbate 60, Sorbitan Isostearate, Sucrose Palmitate, Caprylyl Glycol, Carbomer, *Kappaphycus alvarezii* Extract, Sodium Citrate, Xanthan Gum, Gluconolactone, Dipeptide Diaminobutyroyl Benzylamide Diacetate, Glyceryl Linoleate, Sodium Benzoate, *Crithmum maritimum* Extract, *Prunus amygdalus* Dulcis (Sweet Almond) Oil, Sodium Chloride, Acrylates/C10‐30 Alkyl Acrylate Crosspolymer, *Sigesbeckia orientalis* Extract, Glucose, Sodium Hyaluronate, Potassium Chloride, Potassium Sorbate, Calcium Chloride, Calcium Gluconate, Magnesium Sulfate, Glutamine, Sodium Phosphate, Biotin, Ascorbic Acid, Sodium Acetate, Tocopherol, Lysine HCl, Arginine HCl, Alanine, Histidine HCl, Valine, Leucine, Threonine, Isoleucine, Tryptophan, Phenylalanine, Tyrosine, Glycine, Polysorbate 80, Serine, Cystine, Cyanocobalamin, Glutathione, Asparagine, Aspartic Acid, Ornithine HCl, Glutamic Acid, Nicotinamide Adenine Dinucleotide, Proline, Methionine, Taurine, Hydroxyproline, Glucosamine HCl, Coenzyme A, Sodium Glucuronate, Thiamine Diphosphate, Retinyl Acetate, Inositol, Niacin, Niacinamide, Pyridoxine HCl, Calcium Pantothenate, Riboflavin, Sodium Tocopheryl Phosphate, Thiamine HCl and Folic Acid.

*Imperata cylindrica* Root Extract, *Albizia julibrissin* Bark Extract, *Sigesbeckia orientalis* Extract, *Calanthe discolour* Extract, *Caesalpinia spinosa* Fruit Extract, Adenosine, *Kappaphycus alvarezii* Extract, Dipeptide Diaminobutyroyl Benzylamide Diacetate, and *Crithmum maritimum* Extract being considered its active ingredients, the effects of the eye cream were compared to those of a placebo cream deprived of these ingredients.

### Human skin tissue models

Evaluation of collagen IV and BMP4 expressions was performed using the commercially available human skin tissue EpiDerm‐FT™ model (MatTek Life Sciences, MA, USA). It consists of a fibroblast‐containing dermal layer and an epidermal layer of keratinocytes. Once equilibrated at 37°C with 5% CO_2_, eye cream or placebo topical applications were applied three times over 7 days. Untreated skin served as a negative control.

To evaluate the release of IL‐1α and changes in pigmentation, a commercially available reconstituted human darkly pigmented skin tissue model (MelanoDerm™, MatTek Life Sciences, MA, USA) was used. This model consists of an epidermis constituted of keratinocytes mixed with darkly pigmented melanocytes. After tissue equilibration (37°C, 5% CO_2_), the eye cream or placebo was applied to the surface of the tissue six times over 14 days. Untreated skin models were used as a negative control. A treatment with a 2% Kojic acid solution served as a positive control, as it is well known to inhibit melanin synthesis [19].

### Skin explants

Three types of skin explants were used: abdominal, facial and eyelid NativeSkin® explants (GenoSkin, MA, USA). All were donated surgical discards from females aged 40, 65 and 40, respectively. Tissues were maintained in the recommended media at 37°C with 5% CO_2_. Due to available tissue sizes, analyses were conducted in quadruplicates, triplicates or duplicates, respectively.

The placebo or eye cream was topically applied daily over 5 days for eyelid and facial skin explants. Abdominal explants received four topical applications over 7 days. Untreated samples received no cream application. At the end of the treatment period, tissues were either placed in RNAlater® solution (Life Technologies, Carlsbad, CA, USA) or processed for immunohistochemical analysis.

### Immunohistochemical analysis

Skin tissue models or explants were fixed in 4% paraformaldehyde, embedded in paraffin, and cut into 5‐μm‐thick sections. Primary antibodies recognizing elastin, collagen IV or the bone morphogenetic protein 4 (BMP4) (Abcam, MA, USA) were revealed with goat anti‐rabbit secondary antibodies (Thermo Fisher Scientific, Waltham, MA, USA). All skin sections were also counterstained with DAPI. Labelling was observed using an EVOS FL Auto fluorescent microscope (Thermo Fisher Scientific, Waltham, MA, USA) and analysed using the ImageJ software (Version 1.53 s, NIH, Bethesda, MD, USA). For collagen IV and BMP‐4, results are reported as the mean signal intensity of 5 or 10 images, respectively. Elastin fibre length was determined by measuring the length (in μm) of two fibres per image. Ten images per condition were assessed.

### Gene expression analysis

RNAs were extracted from skin explants (RNeasy® Fibrous Tissue Mini Kit, Qiagen, MD, USA) and quantified (NanoDrop, Thermo Fisher Scientific, MA, USA). cDNA synthesis was performed using the Maxima First Strand cDNA Synthesis kit (Thermo Fisher, MA, USA). Expression analysis of filaggrin (FLG), kallikrein 7 (KLK7), and heparin‐binding EGF‐like growth factor (HB‐EGF) was carried out using Taqman™ gene expression assays (Thermo Fisher, MA, USA) and qPCR (QuantStudio™ 7 Flex, Thermo Fisher). Target gene expression of the eye cream‐treated samples was expressed relative to that of the untreated control. Results and statistical analyses (*t*‐test) were performed using the Thermo Fisher Cloud (Thermo Fisher Scientific). Only results above a 95% confidence level were considered. Results are expressed as the mean fold change of biological triplicates, compared to untreated control.

### IL‐1α and melanin quantification

Following the manufacturer's protocols, Interleukin 1‐alpha (IL‐1α) levels were measured from MelanoDerm™ culture media using ELISA (R&D Systems, MN, USA). Melanin quantification was performed on solubilized MelanoDerm™ tissues by spectrophotometry at an absorbance of 490 nm (SpectraMax® M5, Molecular Devices, CA, USA).

### Non‐enzymatic glycation assay

Non‐enzymatic glycation was evaluated using a modified assay based on previously published approaches [19, 20]. Glycine‐lysine peptide (12 mmol/L GK‐peptide, Chem‐Impex International Inc., Wood Dale, IL, USA) was incubated with 200 mM fructose in 200 mM phosphate buffer (pH 7.0) at 37°C. The effect of 1% (w/v final concentration) placebo or eye cream on the formation of glycation products was monitored by fluorescence quantification at days 0 and 14 (excitation: 355 nm, emission: 440 nm). Control reactions without peptide or sugar were used to establish any background fluorescence.

Non‐enzymatic glycation corresponds to the fluorescence decrease evaluated by subtracting background fluorescence due to reagents and then calculating the change in fluorescence between the initial and final time points. A known inhibitor of glycation, aminoguanidine (0.8 mmol/L) served as a positive control.

### Clinical and instrumental evaluation

A clinical and instrumental evaluation was conducted on 33 healthy women (31 Caucasian and two Asian) aged between 44 and 64 (mean SD = 53 ± 6‐year‐old) and showing slight to moderate ageing signs over the face. The protocol was reviewed and approved by the U.S. Investigational Review Board, Inc. (reference: U.S. IRB2022CP/02), and the study was carried out in Italy. It was conducted in strict conformity with the principles of the Declaration of Helsinki, following Good Clinical Practices, and all subjects gave their written informed consent after receiving detailed information.

For 56 days, subjects self‐applied 2 mg/cm^2^ of cream twice daily (morning and evening) onto their eye contour area. Some short‐term evaluations were also performed after a single application onto the volar side of a randomly selected forearm, the opposite forearm being used as untreated control. Evaluations included skin hydration at the level of the volar forearm quantified using a CM 825 Corneometer® (Courage+Khazaka Electronic GmbH, Germany), transepidermal water loss (TEWL) assessed on the volar forearm with a TM 300 Tewameter® (Courage+Khazaka Electronic GmbH, Germany), and skin profilometry evaluated with the Primos C.R. (Canfield Scientific, NJ, USA). This contactless 3D scanner uses fringe projection to quantify skin surface profiles. The Sv parameter (the depth of the largest valley within a field) was used to evaluate the wrinkle depth of crow's feet, under‐the‐eye wrinkles and frown lines. Skin smoothness in the crow's feet area was assessed using the Ra parameter (the arithmetic average of the absolute value of roughness). Eyelid sagging was quantified with the Rv parameter (maximum valley depth of a profile). The eye‐opening angle was measured by morphometric image analyses of standardized pictures taken with the VisioFace® 1000 D (Courage+Khazaka Electronic GmbH, Germany). Dark circle colour intensity was assessed with a CM‐700D spectrophotometer/colourimeter (Konica Minolta, Japan) based on the ITA° colour parameter.

Clinical evaluations of crow's feet wrinkles, under‐the‐eye wrinkles, frown lines, eyelid sagging, dark circles, and skin brightness were performed by a trained dermatologist. Grading was performed using the photographic scales of Bazin and Doublet [21].

A self‐assessment questionnaire was used to evaluate the perceived product's efficacy. Questions relevant to the product's efficiency were “The product has a visible efficacy on the eye area”, “The product has a visible youth effect”, “The eye look seems less tired”, “The product has a smoothing effect”, “The product seems to reduce/smooth surface/superficial wrinkles”, “The product smoothes the eyelid's crease/wrinkle”, “The product reduces all types of wrinkles around the eye”, “The product seems to attenuate dark circles”, “Eyelids seem tightened”, and “The eye look seems bigger”. Possible answers were “totally agree”, “mostly agree”, “mostly disagree”, or “totally disagree”. “Totally agree” and “mostly agree” were considered positive scores. Time points at which all these assessments were carried out are depicted in Table 1.

**TABLE 1:** Time points of instrumental and clinical evaluations.

Assessment	Baseline (T0)	Short‐term visit (T15 min)	Short‐term visit (T30 min, T2 h, T4 h, T8 h, T24 h)	Intermediate visit (T7 days)	Intermediate visit (T28 days)	Final visit (T56 days)
Skin hydration^a^	X		X			
Transepidermal water loss^a^	X		X			
Skin profilometry (Sv)	X	X		X	X	X
Skin profilometry (Ra, Rv)	X			X	X	X
Eye‐opening angle	X			X	X	X
Dark circle colour	X			X	X	X
Clinical evaluation (crow's feet, under‐the‐eye wrinkles, dark circles, and skin brightness)	X	X		X	X	X
Clinical evaluation (frown lines and glabellar wrinkles, eyelid sagging)	X			X	X	X
Self‐assessment questionnaire				X	X	X

^a^ Assessed on the forearm.

### Statistical analysis

Except for the gene expression analysis, all results from experiments performed on human skin tissue models and skin explants are expressed as mean and standard deviation (SD). Normal data distribution was assessed using the Shapiro–Wilk tests, and statistical analyses were performed using the analysis of variance (ANOVA) followed by Tukey's HSD tests.

Clinical and instrumental evaluation results are reported as the mean and the standard error of the mean (SEM). After verifying normal data distribution with Shapiro–Wilk tests, results from instrumental evaluations were analysed using two‐tailed paired Student's *t*‐tests, or the analysis of variance (ANOVA) and post hoc Tukey–Kramer test. Results from the clinical evaluations were assessed using the nonparametric Friedman's test.

## RESULTS

### Immunohistochemical analysis of collagen IV and BMP4 in a human skin tissue model

Initial experiments to evaluate cream efficacy were performed using a reconstructed skin model. Following topical application of the cream or placebo, immunohistochemical analysis of type IV collagen and BMP4 expression was performed. Collagen IV is expressed at the dermal–epidermal junction and is involved in maintaining eyelid firmness [22]. BMP4 is a multifunctional protein expressed during eye development and opening, as well as in adult skin, to maintain skin homeostasis [23].

**FIGURE 1:**
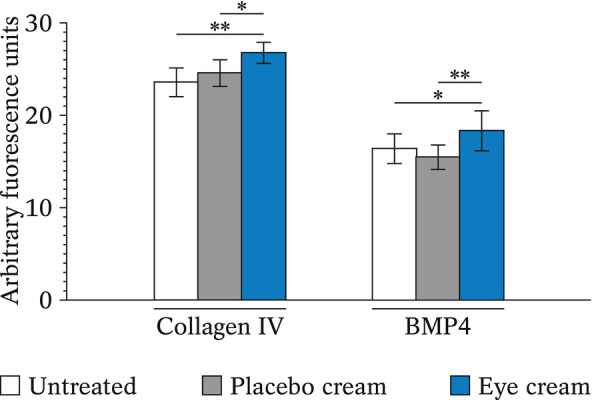
Quantification of the immunohistochemical signal of collagen IV and BMP4 in EpiDerm‐FT™ tissue models untreated or treated with the placebo or the eye cream. Results are from biological quadruplicates. For the statistical analysis, *p < 0.5, **p < 0.01.

Compared to untreated EpiDerm‐FT™ skin models after 7 days (Figure 1), those that received three topical applications of the placebo cream showed no significant variation in the expression of collagen IV (*p* = 0.481) or BMP4 (*p* = 0.493). Only the eye cream had an effect, significantly increasing the expression of collagen IV (+14%, *p* = 0.008 compared to the untreated control; +9%, *p* = 0.048 compared to the placebo) and BMP4 (+12%, *p* = 0.045 compared to the untreated control; +19%, *p* = 0.002 compared to the placebo).

### Immunohistochemical analysis of BMP4 and elastin in skin explants

**FIGURE 2:**
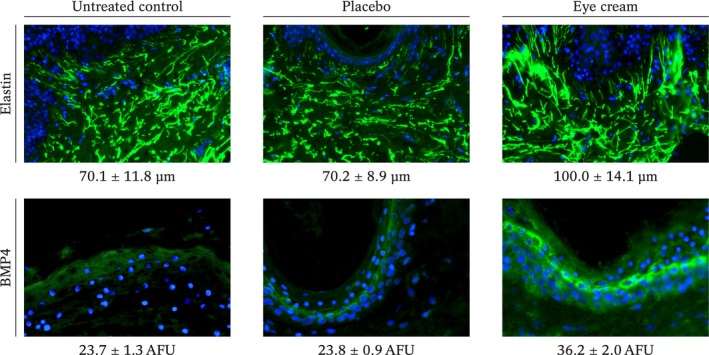
Representative immunohistochemical images of elastin and BMP4 labelling in ex vivo skin explants untreated or treated with the placebo or the eye cream. The number below each image is the mean ± SD of either elastin fibre length (μm) or BMP4 signal intensity (AFU, arbitrary fluorescence units).

We then further investigated BMP4 protein expression in eyelid NativeSkin® explants and elastin expression in abdominal skin explants. Elastin is a major extracellular matrix protein responsible for skin elasticity, so we examined the overall expression and fibre length. Representative immunohistochemical images are presented in Figure 2. Calculation of the average elastin fibre length and quantification of the BMP4 fluorescent signal indicated that the placebo cream had no effect compared to untreated explants for both markers (*p* = 1.000 for elastin, *p* = 0.971 for BMP4). However, the eye cream significantly increases elastin fibre length and BMP4 signal intensity compared to the untreated control and the placebo cream. For elastin, the increase was 43% compared to the untreated control (*p* < 0.001) and 42% compared to the placebo (*p* < 0.001). In the case of the BMP4 signal intensity, the eye cream led to a 53% and 52% increase compared to the untreated control and the placebo, respectively (*p* < 0.001 in both cases).

### Expression analysis of HB‐EGF, Filaggrin and KLK7 in skin explants

The effects of the eye cream on additional skin markers were evaluated using RT‐qPCR performed on RNA extracted from abdominal skin explants (Table 2). The first marker selected was HB‐EGF, a key protein involved in skin homeostasis. Filaggrin and KLK7, two skin barrier‐related markers, were also assessed. Compared to the untreated control, the placebo cream has no effect. Only the eye cream significantly improves the expression of all three markers evaluated.

**TABLE 2:** Fold change in HB‐EGF, Filaggrin and KLK7 expression in placebo‐ and eye cream‐treated abdominal skin explants compared to the untreated skin explant.

Gene	Untreated	Placebo	Eye cream
HB‐EGF	1.00	2.68 (*p* = 0.092)	3.42 (*p* = 0.007)
Filaggrin	1.00	2.17 (*p* = 0.375)	5.33 (*p* = 0.048)
KLK7	1.00	1.42 (*p* = 0.330)	2.52 (*p* = 0.048)

*Note*: Results are the mean of triplicates. The number in between brackets corresponds to the statistical significance resulting from *t*‐tests comparing results to those of untreated samples.

### Quantification of IL‐1α and melanin in human darkly pigmented skin tissue model

Dark circles are an important aspect of periorbital skin ageing. Inflammation and skin pigmentation being linked processes, we interrogated the effect of the eye cream on both by quantifying a key regulator of the inflammatory pathway, IL‐1α, and melanin. In order to maximize the dynamic range for the melanin assay, the experiments were carried out with darkly pigmented MelanoDerm™ tissue models in quadruplicate. Kojic acid, known to decrease melanin synthesis, served as a positive control.

MelanoDerm™ tissues were topically treated six times over 14 days for melanin quantification. As inflammation is an early response to a treatment or stressor, media was collected 24 h after the first application and analysed for secreted IL‐1α expression. Compared to untreated tissue models (26.0 ± 3.9 ng/L of IL‐1α secreted per explant), the placebo cream or the eye cream revealed no significant effect (27.7 ± 10.7 ng/L, *p* = 0.990, and 14.9 ± 6.8 ng/L, *p* = 0.264, respectively), nor did 2% Kojic acid solution (16.1 ± 4.3 ng/L, *p* = 0.088).

Further, none of the cream formulations revealed any significant changes in melanin protein expression. Kojic acid (10.9 ± 2.9 μg of melanin per explant) decreased melanin, as expected, compared to the untreated control (14.2 ± 1.4 μg), but this decrease was at the limit of significance (*p* = 0.085). The placebo cream (14.4 ± 0.8 μg, *p* = 0.997) and the eye cream (14.6 ± 1.2 μg, *p* = 0.986) had no effect.

### Non‐enzymatic glycation

Glycation is a non‐enzymatic reaction occurring between reducing sugars and proteins. It leads to skin damage and the formation of fluorescent or coloured end products. It was assessed using aminoguanidine, a known inhibitor of the reaction, as a positive control. All results are compared to those of the untreated negative control.

**FIGURE 3:**
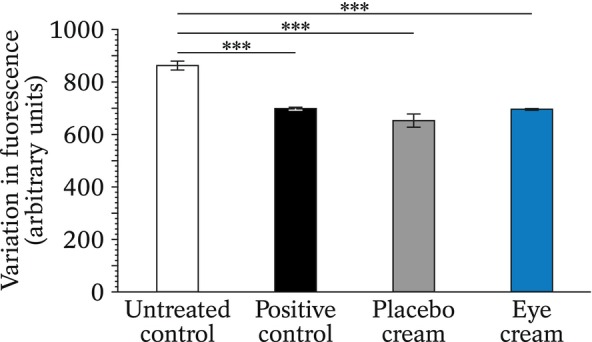
Quantification of the non‐enzymatic glycation in samples untreated or treated with the aminoguanidine‐positive control, the placebo or the eye cream. Results are from triplicate experiments with, for the statistical analysis, ***p < 0.001.

Results (Figure 3) indicated that, as expected, the aminoguanidine‐positive control significantly reduced the glycation reaction (−19%, *p* < 0.001). The placebo, by itself, also led to decreased glycation (−24%, *p* < 0.001), as did the eye cream (−19%, *p* < 0.001). There was no significant difference between the placebo and the eye cream (*p* = 0.998). Both were equally effective at reducing the formation of glycation products.

### Instrumental and clinical evaluation

To understand if the eye cream would provide visible benefits in vivo, we carried out instrumental and clinical evaluations on 33 women who used the eye cream for 56 days. To do so, we compared results at different time points to baseline ones.

The short‐term effect of a single eye cream application on skin water flux was monitored by measuring skin hydration and TEWL (Table 3). Within 30 min, TEWL significantly dropped while skin hydration almost doubled, and these eye cream‐induced changes were still noticeable after 24 h. Besides, the depth of the crow's feet and wrinkles under the eyes were visibly reduced as early as 15 min after a single cream application (Table 4). The reduced visibility of these two wrinkles was demonstrated in the short term and at each time point assessed over 56 days. An increased smoothness of the crow's feet area was also observed after 7 days. The periorbital wrinkle requiring the most time to present reduced depth was the frown lines, where decrease was only significant after 28 days of application.

**TABLE 3:** Results from the short‐term instrumental evaluations onto the volar side of a forearm.

Assessment	T0	30 min	2 h	4 h	8 h	24 h
Transepidermal water loss (g/h/m^2^)	7.9 ± 0.2 –	7.2 ± 0.2 (*p* < 0.001)	7.1 ± 0.2 (*p* < 0.001)	7.1 ± 0.2 (*p* < 0.001)	7.2 ± 0.2 (*p* < 0.001)	7.3 ± 0.2 (*p* < 0.001)
Skin hydration (corneometer units)	25.1 ± 1.0 –	47.3 ± 1.6 (*p* < 0.001)	42.7 ± 1.5 (*p* < 0.001)	39.2 ± 1.4 (*p* < 0.001)	36.3 ± 1.4 (*p* < 0.001)	31.7 ± 1.0 (*p* < 0.001)

*Note*: In between brackets is reported the significance versus T0. Analyses were performed using the analysis of variance (ANOVA) and post hoc Tukey–Kramer test.

**TABLE 4:** Results from the short‐ and long‐term instrumental evaluations.

Assessment	T0	15 min	7 days	28 days	56 days
Crow's feet depth (Sv in μm)	249.2 ± 12.1 –	231.1 ± 11.2 (*p* < 0.001*)	236.0 ± 12.2 (*p* < 0.001*)	229.2 ± 11.7 (*p* < 0.001*)	219.1 ± 10.7 (*p* < 0.001*)
Under the eyes wrinkle depth (Sv in μm)	279.5 ± 12.4 –	261.5 ± 11.6 (*p* < 0.001*)	265.7 ± 11.6 (*p* < 0.001*)	258.6 ± 11.8 (*p* < 0.001*)	249.6 ± 11.1 (*p* < 0.001*)
Frown lines depth (Sv in μm)	344.3 ± 22.2 –	–	335.9 ± 23.1 (*p* = 0.084*)	323.8 ± 21.9 (*p* < 0.001*)	318.0 ± 22.2 (*p* < 0.001*)
Skin smoothness of the crow's feet area (Ra in μm)	38.2 ± 1.7 –	–	35.8 ± 1.8 (*p* < 0.001*)	34.9 ± 1.6 (*p* < 0.001*)	34.3 ± 1.6 (*p* < 0.001*)
Eyelid sagging (Rv in μm)	195.6 ± 8.3 –	–	192.8 ± 7.2 (*p* = 0.646**)	186.3 ± 7.3 (*p* = 0.001**)	181.3 ± 6.9 (*p* < 0.001**)
Eye‐opening angle (°)	35.445 ± 0.898 –	–	36.317 ± 0.819 (*p* = 0.029**)	36.679 ± 0.766 (*p* < 0.001**)	37.400 ± 0.785 (*p* < 0.001**)
Dark circle colour (ITA°)	19.2 ± 1.5 –	–	20.7 ± 1.6 (*p* = 0.004**)	21.0 ± 1.6 (*p* < 0.001**)	21.4 ± 1.7 (*p* < 0.001**)

*Note*: In between brackets is reported the significance versus T0.

* Statistical analysis performed using two‐tailed paired Student's *t*‐tests.

** Statistical analysis performed with the analysis of variance (ANOVA) and post hoc Tukey–Kramer test.

The eye cream also positively impacted other periorbital ageing signs (Table 4). If eyelid sagging required 28 days to decrease, the eye‐opening angle was noticeably increased after only 7 days. As shown by the increased ITA° values, the dark circle colour becomes lighter as soon as after 7 days.

If the improvements mentioned above were detectable instrumentally at early time points, they were also perceivable upon clinical assessment (Table 5). Indeed, assessments by the study dermatologist indicated significant improvement in skin brightness after 15 min, eyelid sagging within 7 days, and dark circles were noticeably less pronounced after 28 days. The cream also leads to a highly significantly reduced scoring of under‐the‐eye wrinkles within 28 days and all other wrinkles after 56 days.

**TABLE 5:** Results from the short‐ and long‐term clinical evaluations.

Assessment	T0	15 min	7 days	28 days	56 days
Crow's feet	3.4 ± 0.2 –	3.2 ± 0.2 (*p* = 0.061)	3.3 ± 0.2 (*p* = 0.173)	3.2 ± 0.2 (*p* = 0.106)	3.2 ± 0.2 (*p* = 0.002)
Under‐the‐eye wrinkles	3.3 ± 0.1 –	3.1 ± 0.1 (*p* = 0.104)	3.1 ± 0.1 (*p* = 0.104)	3.0 ± 0.1 (*p* < 0.001)	2.9 ± 0.1 (*p* < 0.001)
Frown lines / glabellar wrinkles	2.9 ± 0.1 –	–	2.8 ± 0.1 (*p* = 0.751)	2.8 ± 0.2 (*p* = 0.098)	2.7 ± 0.2 (*p* < 0.001)
Eyelid sagging	6.9 ± 0.2 –	–	6.6 ± 0.3 (*p* = 0.018)	6.6 ± 0.3 (*p* = 0.006)	6.4 ± 0.2 (*p* < 0.001)
Dark circles	6.7 ± 0.2 –	6.6 ± 0.2 (*p* = 0.700)	6.5 ± 0.2 (*p* = 0.080)	6.4 ± 0.2 (*p* = 0.004)	6.3 ± 0.2 (*p* = 0.001)
Skin brightness	4.6 ± 0.2 –	5.4 ± 0.2 (*p* = 0.001)	5.2 ± 0.2 (*p* = 0.001)	5.8 ± 0.2 (*p* = 0.001)	6.1 ± 0.2 (*p* = 0.001)

*Note*: In between brackets is reported the significance versus T0.

**FIGURE 4:**
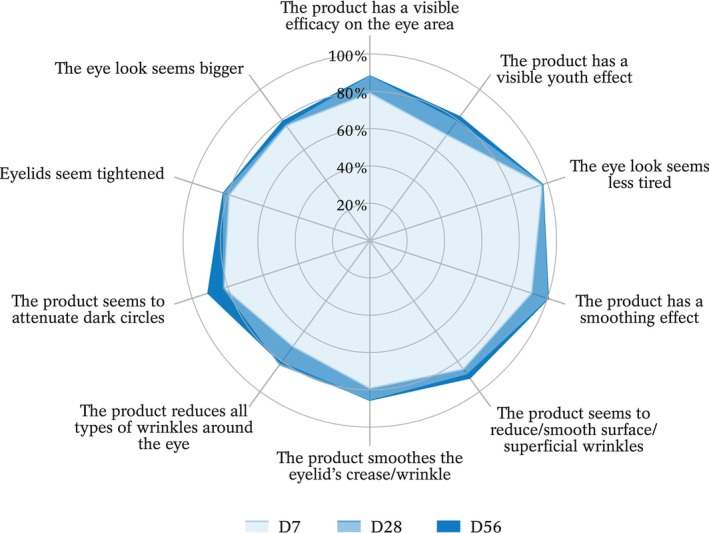
Percentage of “totally agree” or “mostly agree” positive answers in the self‐assessment questionnaire.

Finally, self‐evaluation efficacy by subjects indicated that they very rapidly perceived a positive impact of the eye cream (Figure 4).

## DISCUSSION

The eye and periorbital regions are the first areas of the face undergoing visible age‐related changes, generally around the third decade [3, 4]. This early onset results from a unique combination of thin skin, constant movement, and a high susceptibility to environmental factors. It leads to modifications resulting in dry skin, eye puffiness, wrinkles, and hyperpigmentation, and these are major concerns to many. Achieving perceivable periorbital skin rejuvenation is a complex process that requires addressing all ageing signs. Compounds such as retinoid derivatives, antioxidants, ceramides, hyaluronic acid, etc. individually improve, at least, some of these signs [11, 12, 13, 14, 15, 16, 17, 18]. In this study, we report the early‐, mid‐, and long‐term effects of a new cosmetic formulation combining several ingredients and targeting multiple periorbital ageing signs. To fully understand the potential of this formulation, we carried out clinical and instrumental studies, as well as gained insight into its modes of action by in vitro and ex vivo assessments of relevant molecular markers.

An important aspect of ageing skin is the alteration of barrier function [24]. It is the consequence of profound changes in *stratum corneum* structure, synthesis of its unique lipids, and many facets of its physiology. Changes are manifested as decreases in TEWL and increased skin dryness [24, 25]. The periorbital region is no exception, but a single application of eye cream reduced TEWL and improved skin hydration within half an hour, and the improvement is significant after 24 h. The hyaluronic acid, in concert with additional humectants and emollients present in the eye cream formulation, may partially explain these effects. Hyaluronic acid is well known for its high water affinity and ability to induce water movement from the dermis to the epidermis within minutes [26, 27, 28]. The *Imperata cylindrica* root extract may further boost the hydration and moisturization benefits. The extract has high potassium and dimethylsulfoniopropionate (DMSP) content. The high potassium level should favour increased osmotic pressure of corneocytes due to their Na+/K+ pump, while DMSP should act as an osmoprotectant [29, 30]. Indeed, unpublished preliminary data showed the extract improves skin hydration within 24 h.

TEWL and hydration were not clinically assessed at later time points. Yet, it is reasonable to assume that the cream has long‐term positive effects on the barrier function and skin hydration. This assumption is supported by the higher levels of KLK7 and filaggrin mRNA evidenced by RT‐qPCR after 7 days and four cream applications. KLK7 is a member of a family of secreted serine protease endopeptidases that modulate keratinocyte differentiation. KLK7 is a key enzyme in the process of desquamation, the invisible shedding of corneocytes during skin renewal [31, 32]. Filaggrin is also pivotal to skin barrier function [33]. As a major constituent of corneocytes, its aggregation with keratin contributes to the resistance of the external skin layer [34, 35]. As corneocytes mature, filaggrin fragmentation produces hygroscopic compounds known as the natural moisturizing factors (NMFs), which play a primary role in hydration [36]. The fact that the eye cream leads to higher expression of KLK7 and filaggrin may result in mid‐ and long‐term positive effects on the skin barrier function and hydration.

Another feature improved within minutes by the eye cream is skin profilometry at the level of crow's feet and under‐eye wrinkles. The hyaluronic acid and formulated humectants present in the eye cream could mediate this rapid effect as its topical application is known to induce almost immediate skin plumpness [37]. Among the many ingredients in the composition of the eye cream, two may contribute to the skin profilometry improvement. The galactomannans from the *Caesalpinia spinosa* extract and the galactans from the *Kappaphycus alvarezii* extract are film‐forming oligosaccharides that claim skin‐tensing properties within minutes.

The beneficial effects of the eye cream on wrinkles are not restricted to the short term. It is measurable and significant on overall wrinkle assessment within 7 days and clinically perceivable after 28 or 56 days. Although if it is difficult to relate specific ingredients to this effect, and further work would be needed, we can speculate that several of them may play a role. Dipeptide diaminobutyroyl benzylamide diacetate is a functional analogue of Waglerin‐1, a temple viper toxin that blocks neuromuscular contraction by partially inhibiting neuronal signal transduction [38]. It may lead to decreased crow's feet and forehead wrinkles after 28 days of twice‐daily applications. Another ingredient is, once again, hyaluronic acid. Its repeated application has been shown to reduce wrinkle severity [37]. The mechanism is unclear, as only a subset of small molecular weight hyaluronic acid topically applied enters the dermis [39]. A possible mechanism could involve the interaction of hyaluronic acid with CD44 cell surface receptors, mediating intracellular signalling with the cytoskeleton, EGF, and TGFβ receptors, ultimately stimulating extracellular matrix synthesis [40, 41, 42]. Indeed, wrinkle appearance and severity essentially depend on the breakdown of the extracellular matrix that mainly consists of collagen and elastin [43]. Therefore, any long‐term improvement of wrinkles may involve increased expression and improved morphology of matrix constituents, which we observed with increased elastin fibre length. However, elastin immunostaining shows a positive effect only upon applications of the eye cream, not the placebo cream which also contains hyaluronic acid. Thus, hyaluronic acid might only play a limited role in the wrinkle reduction observed. The last ingredient that may have an effect is the *Albizia julibrissin* bark extract. Unpublished results show that it upregulates CRABP2, the cellular retinoic acid binding protein 2, leading to retinoid‐like effects. If such an effect is confirmed, it would make this extract a good candidate to explain the improvement in elastin. Indeed, retinol does not only stimulate collagen synthesis but also elastin, leading to a global positive effect on the extracellular matrix and wrinkle severity [44].

The most frequent concern among age‐induced changes in the eye region is certainly the drooping of the eyelid. Histological analyses show that age has no noticeable effect on eyelid muscles [45]. Alterations primarily affect the eyelid skin, which presents abnormal elastic fibres and a disrupted collagen network. The eye cream also addresses this concern, as demonstrated by instrumental and clinical evaluation, with significant improvement in eyelid sagging and the eye‐opening angle. As previously mentioned, several eye cream ingredients could positively affect the extracellular matrix and support this improvement. Further, the observed improvement in collagen IV expression by immunostaining may contribute to the reinforcement of the dermal–epidermal junction. This collagen is the most abundant constituent of the basal membrane and controls epidermis development and epidermis–dermis crosstalk [46].

A last important ageing sign of the periorbital region is infraorbital hyperpigmentation. Known as dark circles, it results from the extravasation of haemoglobin breakdown products due to the age‐induced increased permeability of the local vasculature [8]. Changes in the midface soft tissue contribute to its visibility, and hyperpigmentation can be aggravated by inflammatory processes. Clinical and instrumental evaluations of dark circle colour show that the eye cream leads to a lightening of dark circles. Even if it did not induce significant changes in melanin levels or inflammation, the fact that it reduces non‐enzymatic glycation reactions should limit skin damage and the spontaneous formation of pigmented end products. One might have expected an effect on melanogenesis as immunohistological analysis revealed increased BMP4 expression, a known down regulator of melanogenesis, but dark circles are a highly complex phenomenon which is still not completely understood [47, 48].

Finally, immunohistochemical analysis and qRT‐PCR revealed increased expression of two essential skin markers following eye cream application. These markers, HB‐EGF and BMP4, are interesting as they would indicate a global positive effect on skin homeostasis. The role of HB‐EGF in wound healing is well documented [49]. It is a critical mediator of tissue repair and regeneration, positively influencing cellular proliferation, migration, adhesion, and differentiation. If present in all layers of wound margins, its expression is usually restricted to the basal membrane where, in conjunction with its antagonist TGF‐ß, it controls keratinocyte fate [50]. The second marker is BMP4, which is known to play a crucial role in eye development and eyelid opening but also positively affects epidermal cell proliferation and differentiation [23].

## CONCLUSION

The clinical study indicates that the eye cream significantly improves multiple signs of ageing in the periorbital and eye region. In addition to providing very rapid improvement of some of these signs, it also leads to mid‐ and long‐term enhancements that are not only measurable instrumentally but also clinically perceivable. In vitro and ex vivo analyses of relevant markers support and may partially explain these effects. A full understanding of all clinical improvements and the exact role the various ingredients have will require further work.

## FUNDING INFORMATION

The present study was funded by Colgate‐Palmolive Company.

## CONFLICT OF INTEREST STATEMENT

All authors are full‐time employees of Colgate‐Palmolive Company.
